# Lawsone Derivatives as Efficient Photopolymerizable Initiators for Free-Radical, Cationic Photopolymerizations, and Thiol—Ene Reactions

**DOI:** 10.3390/polym13122015

**Published:** 2021-06-20

**Authors:** Christine Elian, Vlasta Brezová, Pauline Sautrot-Ba, Martin Breza, Davy-Louis Versace

**Affiliations:** 1 ICMPE–UMR-CNRS 7182, Université Paris-Est Créteil (UPEC), 2-8 rue Henri Dunant, 94,320 Thiais, France; christine.elian@laposte.net (C.E.); pauline.sautrot@gmail.com (P.S.-B.); 2 Department of Physical Chemistry, Institute of Physical Chemistry and Chemical Physics, Slovak University of Technology in Bratislava, Radlinského 9, SK-812 37 Bratislava, Slovakia; vlasta.brezova@stuba.sk (V.B.); martin.breza@stuba.sk (M.B.)

**Keywords:** lawsone, photopolymerizable photoinitiators, free-radical photopolymerization, cationic photopolymerization, thiol–ene process

## Abstract

Two new photopolymerizable vinyl (2-(allyloxy) 1,4-naphthoquinone, **HNQA**) and epoxy (2-(oxiran-2yl methoxy) 1,4-naphthoquinone, **HNQE**) photoinitiators derived from lawsone were designed in this paper. These new photoinitiators can be used as one-component photoinitiating systems for the free-radical photopolymerization of acrylate bio-based monomer without the addition of any co-initiators. As highlighted by the electron paramagnetic resonance (EPR) spin-trapping results, the formation of carbon-centered radicals from an intermolecular H abstraction reaction was evidenced and can act as initiating species. Interestingly, the introduction of iodonium salt (Iod) used as a co-initiator has led to (1) the cationic photopolymerization of epoxy monomer with high final conversions and (2) an increase of the rates of free-radical polymerization of the acrylate bio-based monomer; we also demonstrated the concomitant thiol–ene reaction and cationic photopolymerizations of a limonene 1,2 epoxide/thiol blend mixture with the **HNQA**/Iod photoinitiating system.

## 1. Introduction

In the past few years, photopolymerization gained an increasing interest and became the cornerstone of many investigations related to microelectronics, adhesives inks, dentistry, and the elaboration of (3D) biomaterials [1]. The success of photochemistry is undoubtedly based on undeniable advantages [2] over the thermal process, e.g., polymerizations under low-temperature conditions without the addition of hazardous solvent, reduced reaction times, the spatial control of the light penetration inside the photosensitive resins, and the capability to adjust overall properties of the photoinduced materials. However, the use of UV-light irradiation still remains an issue of concern in photopolymerization, and alternatives must be found.

In light of these considerations, huge efforts have been made to propose efficient visible-light photoinitiating systems [3,4,5]. These systems can be classified into two distinct categories according to how radicals are generated. Norrish I and II type photoinitiators, acting in the visible range, are well-known to perform free-radical and cationic photopolymerizations. Even if type I photoinitiators, which can cleave under light activation, are extensively used in industries, the irreversible consumption of these kinds of photoinitiators during polymerization is clearly the main drawback. Therefore, scientists have turned their efforts towards developing Norrish type II photoinitiators. In particular, relevant reviews [3,4,6] have described the recent progress photoinitiating strategies envisaged to perform polymerization under light irradiation. Notably, new photocatalytic systems based on three-component photoinitiating systems [7,8] have been put to the forefront without considering environmental issues.

In order to meet environmental challenges, the use of bio-resources appears to be a credible alternative. Surprisingly, even if the use of bio-based monomers [9] for polymer synthesis is now gaining interest from industry and academic researchers, the use of photoinitiators based on natural products is scarce. The first study describing the interest of riboflavin as a photoinitiator for the free-radical polymerization (FRP) of acrylic monomers was published in 1967 [10]. In 2005, Crivello presented the first example of photoinitiating systems based on curcumin [11], a π-conjugated system extracted from the rhizomes of *Curcuma longa*: curcumin was used for the sensibilization of onium salts and the ring-opening polymerization of cyclohexene oxide at 407 nm. According to the extended absorption spectrum of curcumin into the visible range up to 535 nm, and the recent development of household light-emitting diodes (LEDs), Lalevée et al. developed a three-component photoinitiating systems (i.e., curcumin/diphenyliodonium hexafluorophosphate/triphenylphosphine) for dental applications [12]. Curcumin has also been used concomitantly as a photosensitizer for the cationic photopolymerization (CP) of epoxidized soybean oil and as an antibacterial agent under visible-light exposure [13]. Recently, Versace et al. demonstrated the pivotal role of curcumin, judiciously used as a two-photon FRP initiator and as a photogenerator of biocide reactive oxygen species for antibacterial applications [14]. In the same way, Versace et al. used β-carotene as a visible absorbing photosensitizer for the cationic photopolymerization of epoxidized bio-based limonene derivatives for the synthesis of innovative antibacterial materials [15]. Paprika was investigated as a visible photoinitiator [16] when combined with diphenyliodonium salt for the free-radical-induced cationic photopolymerization of a gallic acid derivative under visible-light exposure. Dihydroxyanthraquinone and polyhydroxyanthraquinone derivatives [17,18,19], which are essentially extracted from the madders of the dyers, have been also exploited. Due to their high molar extinction coefficients in the visible range, their capability to initiate FRP, CP, or the thiol–ene process in combination with an appropriate electron acceptor, electron donor, or H-donor molecule, was demonstrated upon visible-light irradiation. Some studies have also investigated the photoreactivity of natural flavones [20] (i.e., quercetin, myricetin, and chrysin) in acrylate and epoxy blend mixture for dental and antibacterial applications [21]. Very recently, vanillin was used as the initial building block to design a new type I cleavable photoinitiator [22] in the UV range. In 2020, two natural dyes [23], namely 5-hydroxy-1,4-naphthoquinone and 2-hydroxy-1,4-naphthoquinone were examined as co-initiators in two- or three-component photoinitiating systems for FRP of acrylates under visible-light irradiation at low light intensity. Despite the high absorption properties of naphthoquinones, low final acrylate conversions were obtained.

The limited studies concerning the use of 2-hydroxy-1,4-naphthoquinone (lawsone) and its low initiating properties for FRP and CP in the visible range prompted us to design new lawsone-derived structures. The novelty of this study is to functionalize lawsone with vinyl or epoxy group to obtain photopolymerizable photoinitiators.

Lawsone (HNQ) is an orange dye derived from the leaves of the henna plant (*Lawsonia inermis*), usually used in textiles, cosmetics, tattoo inks, and in medical domains. Lawsone is also known for its anti-fungal [24,25], anti-corrosive [26], and anti-cancer [27] properties and can be used for the detection of fingerprints [28].

In the present study, two new bio-based photoinitiators derived from lawsone were designed. Their photochemical behavior under irradiation was described in detail by steady-state photolysis and fluorescence experiments. Their photoinitiating properties as a single photopolymerizable photoinitiator and co-initiator combined with electron acceptor (iodonium salt) and/or H-donor thiol molecule for the FRP, CP, and thiol–ene reactions were evaluated by real-time infrared Fourier transform spectroscopy (RT-FTIR) under LEDs@385 and 405 nm. Soybean oil acrylate and limonene 1,2-epoxide were used as completely bio-based monomers for the kinetic studies. Mechanistic investigations and the proposed mechanisms were fully supported by electron paramagnetic resonance (EPR) spin-trapping.

## 2. Experimental Section

### Materials

Metachloroperbenzoic acid (*m*-CPBA), tetraethylammonium bromide (TEAB), 2-hydroxy-1,4-naphthoquinone (lawsone, HNQ), allyl bromide, potassium carbonate (K_2_CO_3_), soybean oil acrylate (SOA), 3,4-epoxycyclohexane)methyl-3,4-epoxycyclohexylcarboxylate (EPOX), limonene 1,2-epoxide (>97%), trimethylolpropane *tris*(3-mercaptopropionate) (trithiol, >95%), 5,5-dimethyl-pyrroline *N*-oxide (DMPO), and *bis*(4-methylphenyl) iodonium hexafluorophosphate (Iod) were purchased from Sigma-Aldrich. Cyclohexane, ethyl acetate, and silica gel were obtained from commercial suppliers. Table 1 displays the chemical structures of the main compounds used in this investigation.

**Synthesis of the allylic derivative (HNQA).** HNQ (435 mg) and tetraethylammonium bromide (TEAB) (81 mg—0.25 mmol—0.1 equivalent) were mixed together with NaOH (100 mg—2.5 mmol—1 equivalent) and K_2_CO_3_ powder (1.382 g—10 mmol—4 equivalents) and transferred to a 30 mL capacity microwave tube, where 6 mL of allyl bromide was added. Reaction was carried out in a microwave reactor under pressure at 75 °C for 1 h. The mixture obtained after reaction in the microwave reactor was transferred to a separatory funnel containing 100 mL of water. The product of interest is extracted three times with 50 mL of diethyl ether. The organic phases are washed with water and dried with magnesium sulfate (MgSO_4_). The solution was filtered into a flask and evaporated. The crude product was purified by column chromatography (silica gel) with the eluent cyclohexane/ethyl acetate in 80/20 proportion. The separation of the compounds was followed by thin-layer chromatography (TLC), and the tubes containing the product of interest were collected in a tared flask, and the contents evaporated. A ^1^H NMR and a ^13^C NMR spectrum were performed to check the structure of the product obtained. The vinylic photoinitiator **HNQA** is a yellow powder. Yield = 48%. **^1^H NMR** (400 MHz, CDCl3) δ 8.12 (d, *J* = 7.2, 1.9 Hz, H3 or H6), 8.07 (d, *J* = 7.2, 1.9 Hz, H3 or H6), 7.72 (m, H4 and H5), 6.16 (s, H9), 6.05 (ddt, *J* = 16.9, 10.9, 5.4 Hz, H12), 5.48 (d, *J* = 16.9, 1.5 Hz, H13a), 5.40 (d, *J* = 10.9 Hz, H13b), 4.61 (d, *J* = 5.4, 1.5 Hz, H11) (see supporting information Figure S1). **^13^C NMR** (100MHz, CDCl3) δ 185.08 (C1 or C8), 180.21(C1 or C8), 159.31 (C10), 134.41 (C4 or C5), 133.47 (C4 or C5), 132.06 (C2 or C7), 131.23 (C2 or C7), 130.59 (C12), 126.83 (C3 or C6), 126.27 (C3 or C6), 120.16 (C13), 110.97 (C9), 70.19 (C11) (see supporting information Figure S2).

**Synthesis of the epoxide derivative (HNQE).** Lawsone vinylic derivative **HNQA** (0.3 g—1.4 mmol—1 equivalent) was dissolved in degassed dichloromethane. *m*-CPBA (2.408 g—14 mmol—10 equivalents) was added four times over 10 h. Each addition must be made after cooling the solution to 0 °C. The solution was then slowly brought to room temperature. After the complete addition of *m*-CPBA, the solution was evaporated. The epoxidation was monitored by thin-layer chromatography (TLC) by regular sampling to observe the disappearance of the **HNQA** stain. The crude product was purified by column chromatography (silica gel) with cyclohexane/ethyl acetate as eluent in a 60/40 ratio. The epoxidized photoinitiator **HNQE** is a yellow powder. Yield = 10%. **^1^H NMR** (400 MHz, CDCl3) δ 8.11 (dd, *J* = 7.2, 1.9 Hz, H3 or H6), 8.06 (dd, *J* = 7.0, 2.0 Hz, H3 or H6), 7.72 (m, H4 and H5), 6.21 (s, H9), 4.29 (dd, *J* = 11.5, 3.0 Hz, H11a), 3.97 (dd, *J* = 11.5, 5.9 Hz, H11b), 3.44 (m, H12), 2.95 (m, H13a), 2.79 (dd, *J* = 4.5, 2.5 Hz, H13b) (see supporting information Figure S3). **^13^C NMR** (100 MHz, CDCl3) δ 185.29 (C1 or C8), 180.16 (C1 or C8), 159.69 (C10), 134.78 (C4 or C5), 133.89 (C4 or C5), 132.32 (C2 or C7), 131.48 (C2 or C7), 127.16 (C3 or C6), 126.62 (C3 or C6), 111.25 (C9), 70.41 (C11), 49.50 (C12), 45.07 (C13) (see supporting information Figure S4).

**Photopolymerization experiments.** The photopolymerization experiments were carried out using real-time Fourier transform infrared spectroscopy (RT-FTIR). The kinetics of photopolymerization were carried out with a time-resolved Fourier transform infrared spectrometer (JASCO 4100 series) to evaluate the absorbance decrease of acrylate, epoxy, vinyl, and thiol functional groups at 1636 cm^−1^, 842 cm^−1^, 1645 cm^−1^, and 2560 cm^−1^, respectively. Formulations were deposited on a BaF_2_ pellet and were irradiated in laminate (with the addition of a transparent polypropylene film on the top of the photosensitive layer) or under air upon LED@385 and @405nm irradiation at room temperature.

**Steady-State photolysis**. Steady-state photolysis of HNQ, HNQ/Iod, **HNQA**, **HNQA**/Iod, **HNQE,** and **HNQE**/Iod systems was conducted by UV-visible spectroscopy (Cary 60 UV-visible spectrometer from Agilent Technologies) in acetonitrile, under air, in a 1 cm quartz cell, and upon LED@385 nm exposure (44 mW/cm^2^).

**Irradiation Sources.** Thorlabs light-emitting diodes from THORLABS (LEDs@385 and 405 nm) were used to initiate cationic and free-radical photopolymerizations. Intensities of LED@385 and 405 nm were 44 mW/cm^2^ and 60 mW/cm^2^, respectively.

**Fluorescence Experiments.** Fluorescence experiments were performed and collected at room temperature with a FluoroMax+ spectrofluorometer from HORIBA. For fluorescence measurements, ACN solution of HNQ, **HNQA,** or **HNQE** were excited at λ_exc_ = 330 nm. The fluorescence quenching of the lawsone derivatives was evaluated after the addition of a gradual concentration of Iod.

**Cyclic Voltammetry.** Cyclic voltammetry measurements of HNQ, **HNQA,** and **HNQE** were performed at room temperature under argon atmosphere according to a previously described method^18^. Glassy carbon, saturated calomel, and gold wire electrodes were used as the working electrode, reference electrode, and counter electrode, respectively. For cyclic voltammetry measurements, the three electrodes were immersed in an ACN solution of HNQ, **HNQA,** or **HNQE** containing a supporting electrolyte solution of tetraethylammonium tetrafluoroborate at 10^−3^ mol. L^−1^. The free energy change ΔG_eT_ for an electron transfer reaction between **HNQA**, or **HNQE** and Iod can be evaluated from the classical Rehm−Weller equation [29].

**Electron paramagnetic resonance** (EPR) **spin-trapping**. The X-band cw-EPR spectra (modulation frequency of 100 kHz) were monitored with the EMX*plus* spectrometer (Bruker) equipped with the High Sensitivity Probe-Head (Bruker) in a small quartz flat cell (Wilmad-LabGlass, WG 808-Q). The solutions prepared in acetonitrile and saturated with argon were irradiated at 295 K directly in the EPR resonator utilizing a LED@400 nm source (*λ*_max_ = 400 nm; Bluepoint LED, Hönle UV Technology). The EPR spectra were recorded in situ during/after a defined exposure as described previously in Reference [18]. The *g*-factors were determined with an uncertainty of ± 0.0001 exploiting a nuclear magnetic resonance teslameter (ER 036TM, Bruker) and integrated frequency counter. The experimental EPR spectra were analyzed by the WinEPR software (Bruker), and the calculations of simulated spectra were performed with the EasySpin toolbox working on the MatLab^®^ platform [30]. The standard EPR spectrometer settings were as follows: microwave frequency, ~9.43 GHz; microwave power, ~10.5 mW; center field, ~336.0 mT; sweep width, 2–8 mT; gain, 2.00 × 10^5^–5.02 × 10^4^; modulation amplitude, 0.025 mT–0.10 mT; sweep time, 45 s; time constant, 10.24 ms or 20.48 ms; the number of scans, 5 or 10. Sample compositions comprising the reagents are discussed in the text.

**Computational method.** Standard MP2 method [31,32] and cc-pVTZ basis sets for all atoms [33] were used for geometry optimizations of the anionic compounds under study in acetonitrile in doublet ground spin states. Solvent effects were approximated by the Solvation Model based on the Density [34] (SMD) continuum model. The stability of the optimized structures was confirmed by vibrational analysis (no imaginary vibrations). Subsequently, their isotropic hyperfine coupling constants in acetonitrile were calculated by DFT method using standard B3LYP hybrid functional [35], EPR-III basis sets for all atoms [36], and SMD solvent model [34]. All calculations were performed using the Gaussian16 [37] program package.

## 3. Results and Discussion

**Scheme 1:**
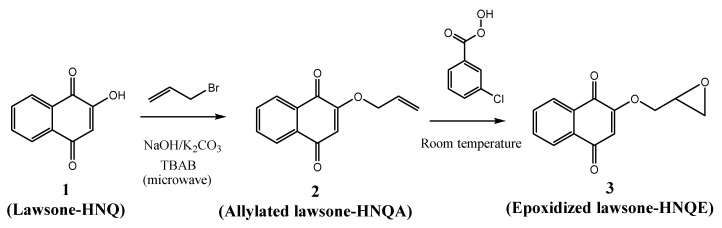
Synthesis of the lawsone derivatives.

**Synthesis of the lawsone derivatives.** Allylated (**HNQA**) and epoxidized (**HNQE**) lawsone were synthesized from lawsone in a one or two-step procedure, following the procedure described in Scheme 1. The synthesized molecules have been fully characterized by NMR spectroscopy (see supporting information). Allylation of lawsone (HNQ) is expected according to a previously reported synthesis [38] using allyl bromide (co-reactant) with NaOH/K_2_CO_3_ as a base catalyst. Prior to the addition of the allyl bromide, a deprotonation of the alcohol group of HNQ occurs and leads to an alcoholate formation, which ensues a nucleophilic SN2 type reaction with allyl bromide. The purification of the crude on silica gel chromatography led to the expected allylated lawsone with a yield of 48%. Interestingly, new proton signals attributed to the vinyl function [38,39] of **HNQA** appear accordingly at 6.1 ppm (H_2_C=C**H**-) and at 5.5 ppm (**H_2_**C=CH-). The oxidation of **HNQA** was performed in degassed dichloromethane at room temperature with *m*-CPBA to convert the allyl bonds into epoxides [39]. After purification of the crude, **HNQE** was obtained with a 10% yield. A complete disappearance of the allyl protons of **HNQA** is observed, leading to the apparition of new signals, which are attributed to the protons of the **HNQE** epoxy group between 2.8 and 3.5 ppm [39].

**Steady-state photolysis and fluorescence.** The UV-vis absorption spectra and the molar extinction coefficient of the lawsone derivatives are displayed in Figure S5. The photophysical properties of the lawsone derivatives are gathered in Table 2. Lawsone derivatives exhibit high molar absorption coefficients in comparison with common visible-light photoinitiators [40] (e.g., camphorquinone or benzophenone) and display a low absorption band in the visible range. This makes lawsone derivatives probable visible photoinitiators in the UV-vis range. The overall photoreactivity of the lawsone derivatives with oxidative compounds was first studied by steady-state photolysis and fluorescence experiments.

**Figure 1:**
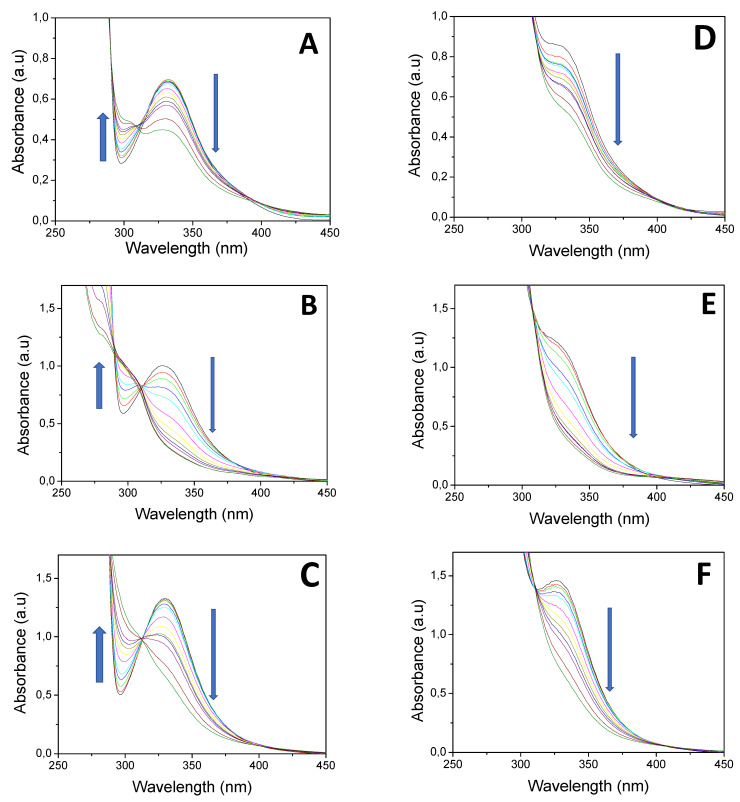
Steady-state photolysis of (**A**) HNQ, (**B**) **HNQA**, (**C**) **HNQE**, (**D**) HNQ/Iod, (**E**) **HNQA**/Iod, and (**F**) **HNQE**/Iod upon LED@385 nm exposure (44 mW/cm^2^). UV−vis spectra were recorded at different irradiation times under air after 420 s of irradiation. [HNQ] = 2.1 × 10^−4^ M, [**HNQA**] = [**HNQE**] = 5 × 10^−4^ M, [Iod] = 1.7 × 10^−3^ M.

Photolysis of HNQ, **HNQA,** and **HNQE** upon LED@385 nm exposure, without and with Iod, are given in Figure 1A−F. Under light irradiation, the absorbance of HNQ, **HNQA**, and **HNQE** alone (Figure 1A,B,C) decreases, and an isosbestic point is clearly observed for each photoinitiating system, suggesting the formation of a new species. Radical species resulting from the irradiation of the naphthoquinone derivatives are likely to appear as EPR spin-trapping could further demonstrate. New carbon-centered radicals are expected to be formed through an H-abstraction reaction as previously described with thioxanthone derivatives [41]. Surprisingly, the absorbance decrease of the lawsone derivatives was not accelerated when adding Iod (Figure 1D–F). Quenching of fluorescence was performed to learn about the reactivity of **HNQA**, and **HNQE** excited singlet states toward the addition of Iod. Fluorescence spectra of **HNQA** and **HNQE** were recorded in ACN (see Figures S6 and S7, respectively), and the maximum emission wavelength was observed at 410 and 411 nm, respectively. A quenching rate of fluorescence was observed for **HNQA** and **HNQE** in the presence of Iod (Figures S6 and S7), with K_SV_^Iod^ (**HNQA**) = 4215 M^−1^ and K_SV_^Iod^ (**HNQE**) = 110 M^−1^. The negative ΔG_eT_(S) values calculated from the Rehm–Weller equation^29^ and the potentials of oxidation (see Figure S8 for E_ox_(**HNQA**) and Figure S9 for E_ox_(**HNQE**)) demonstrate that a thermodynamically favorable electron transfer reaction between the excited singlet states of **HNQA** or **HNQE** and Iod (Table 2) may occur.

**Figure 2:**
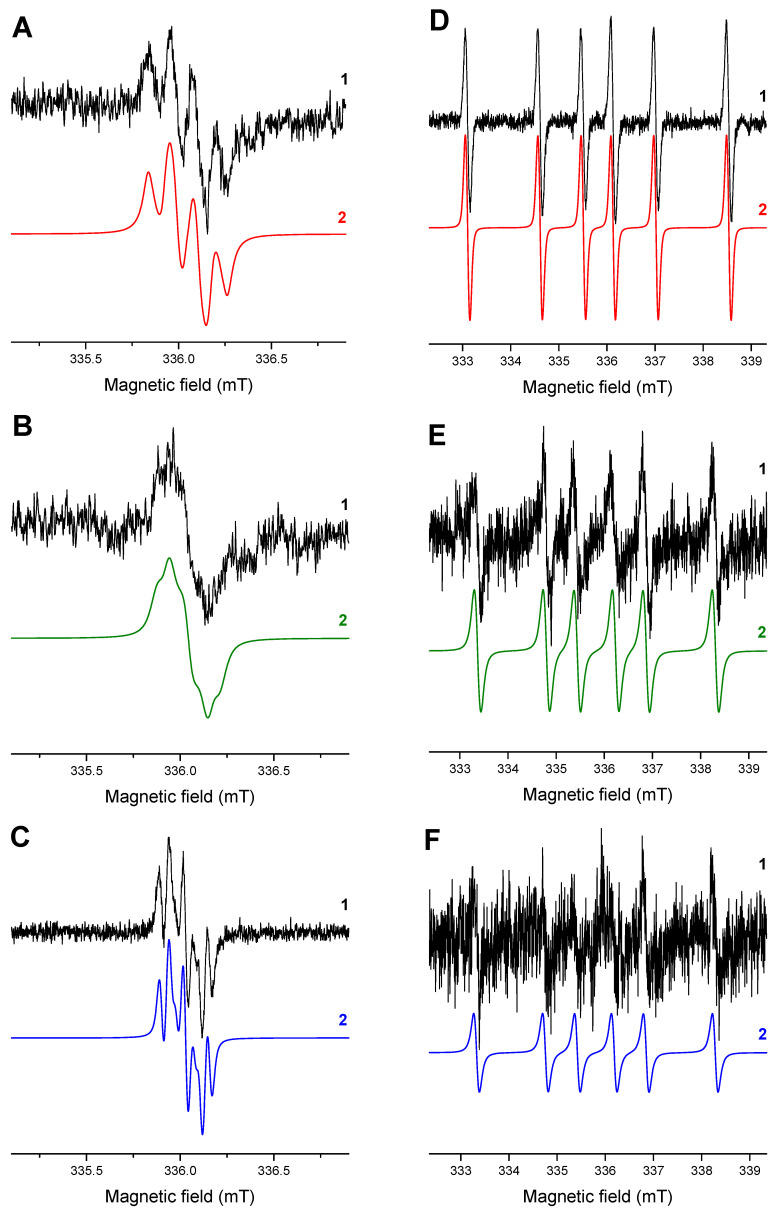
The normalized experimental (1) and simulated (2) EPR spectra obtained upon continuous in situ LED@400 nm exposure: (**A**) HNQ, (**B**) HNQA, (**C**) HNQE, (**D**) HNQ/DMPO, (**E**) HNQA/DMPO, and (**F**) HNQE/DMPO in acetonitrile under argon. Modulation amplitude: 0.1 mT (**A**,**B**) and 0.025 mT (**C**–**F**).

**EPR experiments**. The photoexcitation of HNQ in the deoxygenated acetonitrile solution under given experimental conditions resulted in the generation of EPR signal (*g* = 2.0044) shown in Figure 2A, assigned to the corresponding radical anion [42] (HNQ^•−^). Due to its low intensity, it was not possible to acquire EPR spectra with the lower modulation amplitude, which prevented us from obtaining the better resolved hyperfine structure. Consequently, the simulation interpretation of the experimental spectrum of HNQ^•−^ represents the hyperfine interaction of four dominant hydrogen nuclei with the unpaired electron. As the EPR spectra of HNQ^•−^ found in the literature are significantly affected by the experimental conditions [43], the assignment of hyperfine couplings of individual hydrogen atoms is based on the theoretical data evaluated by the B3LYP/EPR-III/SMD (acetonitrile) calculations (Table 3). HNQ^•−^ contains three oxygen sites where, in principle, the hydroxyl H atom can be located. MP2/cc-pVTZ/SMD (acetonitrile) geometry optimizations of the corresponding structures confirmed the highest stability of the 2-hydroxy isomer, whereas the concentrations of the 1- and 4-hydroxy isomers are vanishing because their Gibbs free energies at 298 K are higher by 109.1 and 33.1 kJ/mol, respectively. While a low-intensity EPR spectrum was obtained during irradiation of **HNQA** in acetonitrile under given experimental conditions (*g* = 2.0047; Figure 2B), the photoactivation of **HNQE** in acetonitrile upon LED@400 nm is analogous, but a better-resolved EPR spectrum is achieved (*g* = 2.0047; Figure 2C); the signals are attributed to the corresponding radical anions of **HNQA** and **HNQE**, respectively (Table 3).

The continuous in situ LED@400 nm irradiation of HNQ/acetonitrile/argon in the presence of DMPO spin trap resulted in the immediate generation of six-line signal (Figure 2D) with the spin-Hamiltonian parameters characteristic for the DMPO-adduct with a ketyl radical [44] (^•^C(OH)R_1_R_2_, Table 4). On the other hand, the irradiation of deoxygenated acetonitrile solutions of **HNQA** and **HNQE** containing DMPO led to the formation of ^•^DMPO-CR adduct (Figure 2E,F), which correspond to the carbon-centered radical in α-position of the vinyl or epoxy group of **HNQA** or **HNQE**, respectively, and formed after an intermolecular H-abstraction reaction.

**Figure 3:**
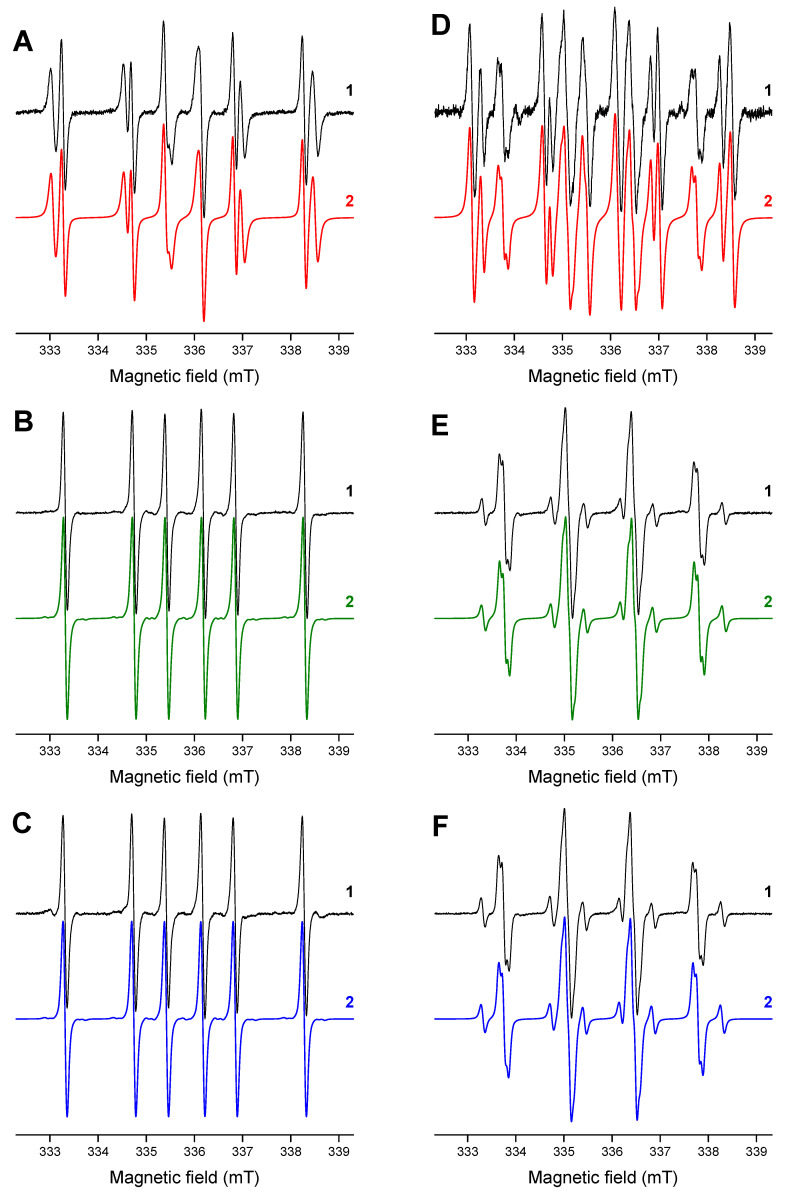
The normalized experimental (1) and simulated (2) EPR spectra obtained upon in situ LED@400 nm exposure of 2-substituted naphthoquinone derivatives in acetonitrile under argon in the presence of DMPO spin trap: (**A**) HNQ/Iod, (**B**) **HNQA**/Iod, (**C**) **HNQE**/Iod, (**D**) HNQ/Iod/TT, (**E**) **HNQA**/Iod/TT, and (**F**) **HNQE**/Iod/TT. Irradiation time: 450 s (**A**–**C**) and 225 s (**D**–**F**). Modulation amplitude of 0.025 mT.

The effective generation of DMPO-adducts with ketyl and phenyl(4-methyl) radicals is well evidenced upon irradiation of HNQ/Iod/DMPO/acetonitrile/argon solutions, also reflecting the efficient electron transfer from the photoexcited HNQ to Iod (Figure 3A). The electron transfer reaction from the photoactivated **HNQA** and **HNQE** molecules to Iod producing phenyl(4-methyl) radicals upon LED@400 nm exposure is demonstrated in Figure 3B,C: a well-resolved EPR signal of the DMPO-phenyl(4-methyl) adduct [45] is only observed. The EPR spectra measured upon in situ LED@400 nm irradiation of 2-substituted naphthoquinone derivatives in deoxygenated acetonitrile solutions containing DMPO/Iod/TT are shown in Figure 3D–F. The EPR spectrum obtained under given experimental conditions for HNQ represents a superposition of three individual DMPO-adducts: (*i*) ^•^DMPO-C(OH)R_1_R_2_; (*ii*) ^•^DMPO-phenyl(4-methyl); and (*iii*) ^•^DMPO-SR; the spin-Hamiltonian parameters are gathered in Table 4. Surprisingly, EPR spectra measured for the photoactivated **HNQA** and **HNQE** under identical experimental conditions (DMPO/Iod/TT/acetonitrile/argon) demonstrated the presence of two latter signals only (^•^DMPO-phenyl(4-methyl)^17^ and ^•^DMPO-SR^17^).

**Scheme 2:**
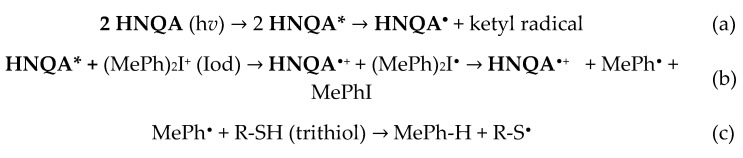
Photoinduced generation of reactive species from **HNQA**/Iod/trithiol photoinitiating system.

According to the EPR results, various active radical species are produced under light irradiation of the lawsone derivatives photoinitiating systems, i.e., **HNQA** (or **HNQE**)/Iod and **HNQA** (or **HNQE**)/Iod/trithiol, and a proposed mechanism is described in Scheme 2 with **HNQA** (Equations (a)–(c)).

**Figure 4:**
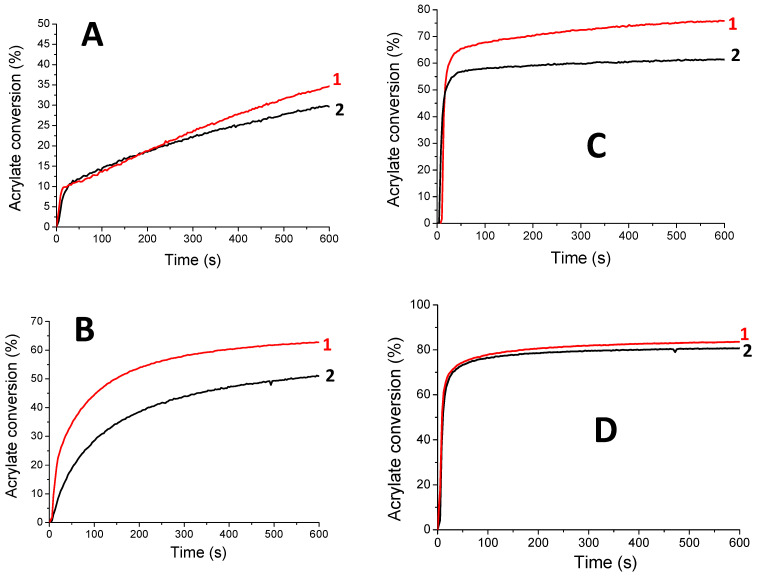
Kinetic profiles for the free-radical photopolymerization of SOA with the following initiating systems: (**A**) **HNQA** (0.5 wt%), (**B**) **HNQE** (0.5 wt%), (**C**) **HNQA**/Iod (0.5/2% *w*/*w*), and (**D**) **HNQE**/Iod (0.5/2% *w*/*w*) upon (1) LED@405 nm and (2) LED@385 nm in laminate.

**Free-radical polymerization of SOA.** HNQ, **HNQA,** and **HNQE** have been first used as a one-component photoinitiating system without the addition of any co-initiators (Figure 4A,B). The free-radical polymerizations of SOA were followed in laminate by RT-FTIR under LEDs@405 nm and 385 nm irradiation. The final acrylate conversions of SOA are displayed in Table 5. Interestingly, **HNQA** and **HNQE** alone can initiate the FRP of SOA with interesting acrylate conversions. These results confirm the previous EPR results and highlight the capability of the resulting carbon-centered radicals (Figure 2E,F) to initiate FRP of acrylate monomer. This is particularly interesting in terms of the green chemistry approach as only one component photoinitiating system is used, reducing the carbon atoms consumption. Not surprisingly, when **HNQA** (or **HNQE**) is combined with Iod, the final acrylate conversions and the rates of polymerization (Rp/[M0]) strongly increase whatever the LEDs used, as described in Table 5 and Figure 4C,D. This confirms the formation of phenyl-derived radicals as highlighted by EPR spin-trapping (Figure 3B,C); such radicals can also initiate the FRP of SOA. Unfortunately, HNQ and HNQ/Iod systems cannot initiate FRP as 1) the anion radical HNQ^•^^−^ is not an effective initiation of radical species, and 2) even if phenyl-derived radicals are generated from the photolysis of HNQ/Iod (Figure 3A), ketyl radicals, also produced by the photolysis of HNQ/Iod, can act as a terminating agent of the polymerization reaction [46].

**Figure 5:**
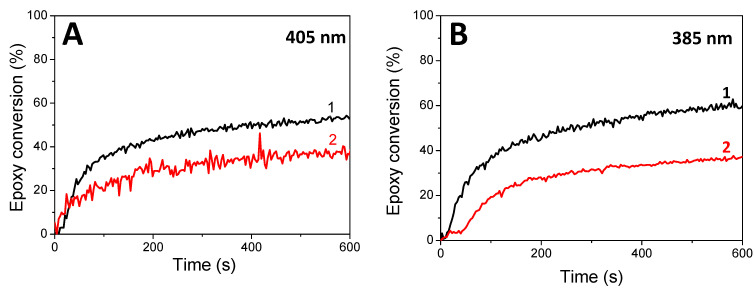
Kinetic profiles for the cationic photopolymerization of EPOX under LEDs@ (**A**) 405 nm and (**B**) 385 nm irradiation with (1) **HNQA/**Iod (0.5/2% *w*/*w*) and (2) **HNQE**/Iod (0.5/2% *w*/*w*) under air.

**Cationic photopolymerization of EPOX.** The kinetics of EPOX polymerization (epoxy conversion vs. irradiation time) in the presence of **HNQA** (or **HNQE**)/Iod photoinitiating systems under different LEDs irradiation under air are described in Figure 5. HNQ/Iod has been used as a reference photoinitiating system for comparison. When Iod is used alone, no photopolymerization of EPOX occurs under LED irradiation (385 and 405 nm) because Iod shows a maximum absorption band around 250 nm. Cationic photopolymerizations of EPOX successfully take place (Figure 5) when using both photoinitiating systems (**HNQA** (or **HNQE**)/Iod) under LEDs@385 and 405 nm exposure. Accordingly, the final conversions of epoxy groups reach 50% after 600 s of irradiation without any inhibition period (Figure 5). These results are in accordance with the production of photoacids (according to the rhodamine B method^21^, Figure S10), responsible for the cationic photopolymerization of EPOX. In comparison, the HNQ/Iod photoinitiating system was also tested, and no cationic photopolymerization was observed: hydroxyl group of HNQ may be responsible for the termination of the growing polymer chain during photopolymerization, thus inhibiting the cationic photopolymerization of EPOX. As a comparison, and under the same irradiation conditions, two well-known type II photoinitiators (i.e., BP and CQ) fail to initiate the photopolymerization of EPOX when coupled with Iod, therefore demonstrating the superior initiating properties of **HNQA** (or **HNQE**)/Iod systems. Therefore, only **HNQA** (or **HNQE**)/Iod systems will be considered in the following study.

**Figure 6:**
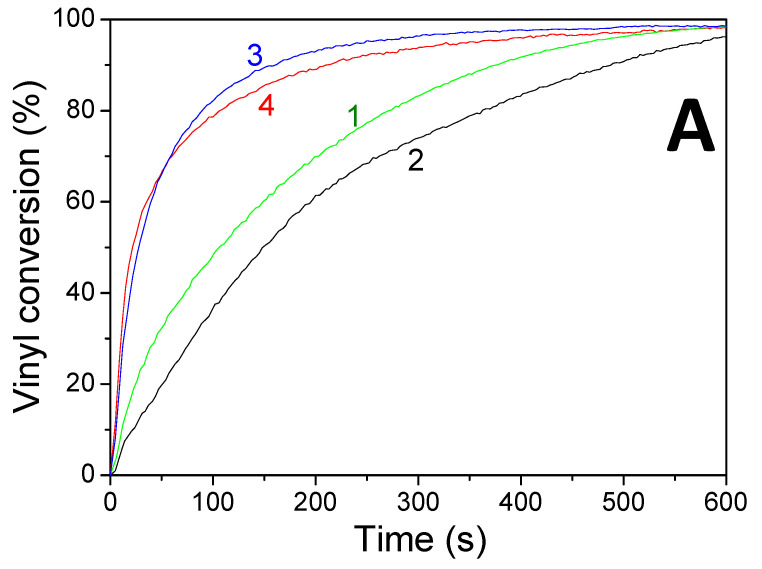

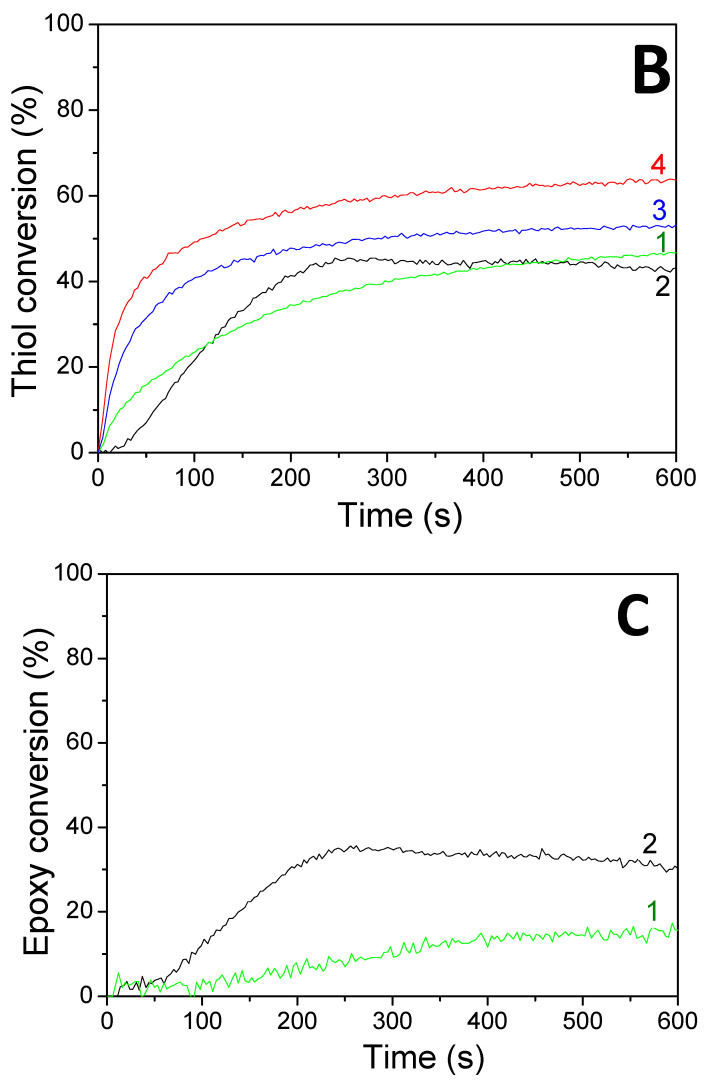
Kinetic profiles for the thiol–ene and cationic photopolymerization of limonene 1,2 epoxide (50wt%)/trithiol (50wt%) blend mixture with **HNQA**/Iod (0.5/2%, *w*/*w*) photoinitiating system, upon LEDs@ (1) 385 nm and (2) 405 nm irradiation under air; and in laminate under LEDs@ (3) 385 nm and (4) 405 nm. (**A**) Vinyl conversions, (**B**) thiol conversions, and (**C**) epoxy conversions.

**Figure 7:**
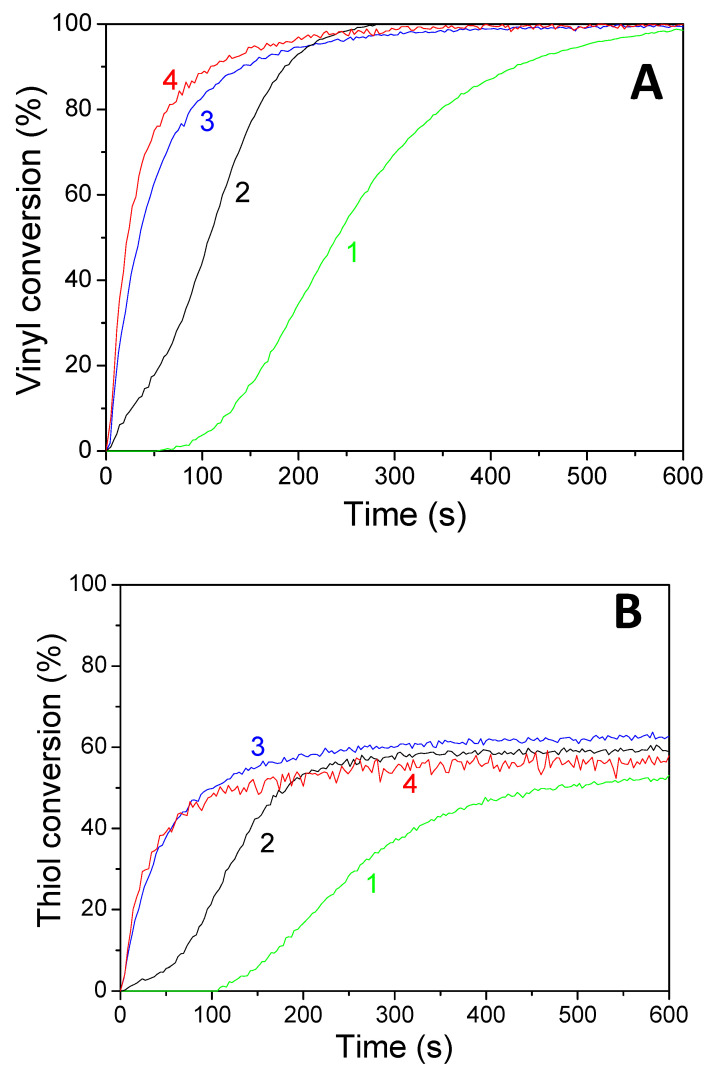
Kinetic profiles for the thiol–ene reaction of limonene 1,2 epoxide (50 wt%)/trithiol (50 wt%) blend mixture with **HNQE**/Iod (0.5/2%, *w*/*w*) photoinitiating system, upon (1) LED@385 nm and (2) LED@405 nm under air; and in laminate under (3) LED@385 nm and 4) LED@405 nm. (**A**) Vinyl conversions and (**B**) thiol conversions.

Concomitant cationic photopolymerization and thiol–ene reactions of a limonene 1,2 epoxide/trithiol blend with HNQA/Iod (Figure 6) and HNQE/Iod (Figure 7) photoinitiating systems. The vinyl, thiol, and epoxy groups were followed under air and in laminate under LEDs@385 and 405 nm. Effective kinetic profiles for the concomitant thiol−ene process and cationic photopolymerization of the limonene 1,2 epoxide/trithiol blend mixture are observed under LEDs@385 and 405 nm exposure with HNQA/Iod (Figure 6) and HNQE/Iod systems (Figure 7). Interestingly, the final vinyl bond conversions reach 100% after 600 s of LED exposure in laminate or under air (Table 6), and still remain higher than those of thiol, thus demonstrating that the limonene 1,2 epoxide polymerization occurs through a thiol–ene reaction (Equation (1)) and albeit to a lesser extent in a radical process. Indeed, according to EPR results, the phenyl(4-methyl) radicals that are produced from the photolysis of HNQA (or HNQE)/Iod systems (Figure 3B,C) may add to the vinyl group of limonene 1,2 epoxide or favor a H-abstraction reaction to generate thiyl radicals according to Equation (c). Under air, the thiyl and phenyl(4-methyl) radicals react with dissolved oxygen to form peroxide radicals (ROO•, see Equation (2)) responsible for the slowing down of the limonene 1,2 epoxide polymerization. Therefore, the rates of polymerization are twice as high in laminate than under air. The following proton transfer reaction between ROO• and trithiol (Equation (2)) regenerates thiyl radicals that can add to the vinyl group, explaining that the thiol–ene reaction occurs under air whatever the LED used. Surprisingly, the cationic photopolymerization of limonene 1,2 epoxide occurs only with the HNQA/Iod photoinitiating system (Figure 6C). This could be easily explained by the fact that the polymerization of limonene 1,2 epoxide/trithiol blend mixture with HNQE/Iod system is faster than the one involving HNQA/Iod, increasing thus the viscosity of the formulation, reducing the diffusion of cationic species in the blended mixture, therefore inhibiting the cationic ring-opening polymerization of limonene 1,2 epoxide.

RS^•^ + R’–CH =CH_2_ (limonene 1,2-epoxide) → R’–CH^•^–CH_2_–SR + RS–H → R’–CH_2_–CH_2_–SR + RS^•^ (1)

R^•^ + O_2_ → ROO ^•^ + RS–H → ROOH+ RS^•^ (2)

## 4. Conclusions

Two new photopolymerizable lawsone derivatives bearing vinyl (**HNQA**) or epoxy (**HNQE**) groups have demonstrated effective initiating properties for the free-radical photopolymerization of a bio-based acrylate (SOA) when used alone. According to EPR investigations, the carbon-centered radicals in α-position of the vinyl or epoxy group of **HNQA** or **HNQE**, respectively, and formed through an intermolecular H-abstraction reaction, can initiate the FRP of SOA under LED@385 and 405 nm irradiation. Furthermore, the addition of Iod, an electron acceptor and co-initiator of the lawsone derivatives, leads to effective free-radical and cationic photopolymerizations of acrylate and epoxy monomers, respectively, under visible-light irradiation. Notably, the rates of free-radical polymerization with Iod strongly increase whatever the LEDs are used. We also demonstrated that the **HNQA**/Iod system could promote both the concomitant thiol–ene reaction and the cationic photopolymerization of a limonene 1,2 epoxide/trithiol blend mixture upon LED@385 and 405 nm exposure with final vinyl bond conversions of around 100% even under air. On the contrary, the thiol–ene reaction toward limonene 1,2 epoxide double bonds is so effective using the **HNQE**/Iod system that the diffusion of cationic species is reduced in the blended mixture, thus inhibiting the cationic ring-opening polymerization of limonene 1,2 epoxide.

## Figures and Tables

**Table 1:** Chemical structures of the monomers and photoinitiating systems used in this study.

Name	Chemical Structure
2-hydroxy-1,4-naphthoquinone (HNQ, Lawsone)	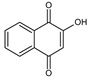
2-(allyloxy) 1,4-naphthoquinone (HNQA)	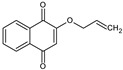
2-(oxiran-2yl methoxy) 1,4-naphthoquinone (HNQE)	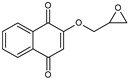
3,4- epoxycyclohexane)methyl-3,4-epoxycyclohexylcarboxylate (EPOX)	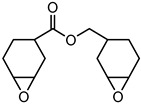
*Bis*(4-methylphenyl) iodonium hexafluorophosphate (Iod)	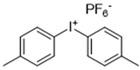
Trimethylolpropane *tris*(3-mercaptopropionate) (trithiol	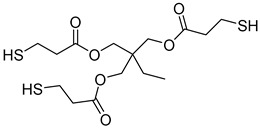
Soybean oil acrylate (SOA)	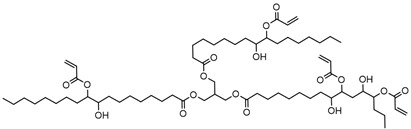

**Table 2:** Photophysical properties of HNQ, **HNQE,** and **HNQA** in acetonitrile.

	λ_max_ (nm)	λ_max_^Em^ (nm)	E_S_ (eV)	E_ox_ (V/SCE)	E_Red_ (V/SCE)	ΔG_eT_ (S) (eV)
HNQ	335	-	-	+1.82		-
**HNQE**	330	411	3.024	+1.44		−1.372
**HNQA**	329	410	3.016	+1.33		−1.499
Iod					−0.2	

**Table 3:** The hyperfine coupling constants of 2-substituted naphthoquinone radical anions calculated at B3LYP/EPR-III/SMD (acetonitrile) level of theory in comparison with those elucidated from the simulations of experimental EPR spectra (*in italic*).

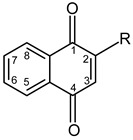	*a* (mT)					
	H(2)	H(3)	H(5)	H(6)	H(7)	H(8)
HNQ^•−^ (R=hydroxy)	−0.064 (OH) *0.039*	−0.154 *0.142*	0.002 *<0.02*	−0.123 *0.114*	−0.040 *<0.02*	−0.076 *0.091*
HNQA^•−^ (R=allyloxy)	−0.014, 0.023 (CH_2_) *<0.02*	−0.235 *0.098*	−0.015 *<0.02*	−0.086 *0.083*	−0.055 *0.057*	−0.045 *0.053*
HNQE^•−^ (R=oxiran-2-yl-methoxy)	−0.012, 0.030 (CH_2_) *<0.02*	−0.238 *0.083*	−0.015 *<0.02*	−0.086 *0.073*	−0.054 *0.054*	−0.045 *0.044*

**Table 4:** Spin-Hamiltonian parameters of DMPO spin-adducts evaluated from the simulation spectra measured in acetonitrile at 295 K.

Radical Added to DMPO Spin Trap	*a*_N_, mT	*a*, mT	*g*-Factor
^•^C(OH)R_1_R_2_	1.515 ± 0.005	2.411 ± 0.005 (H^β^)	2.0059 ± 0.0001
^•^CR	1.436 ± 0.003	2.090 ± 0.010 (H^β^)	2.0060 ± 0.0001
^•^Phenyl(4-methyl)	1.438 ± 0.005	2.116 ± 0.005 (H^β^) 0.773 ± 0.03 (2 × ^13^C), 0.604 ± 0.01 (^13^C), 0.532 ± 0.01 (^13^C)	2.0060 ± 0.0001
^•^SR’	1.369 ± 0.003	1.308 ± 0.003 (H^β^) 0.080 ± 0.003 (H^γ^) 0.072 ± 0.003 (H^γ^)	2.0062 ± 0.0001

**Table 5:** Final acrylates conversions (determined by IR) in laminate upon LED@385 nm (44 mW/cm^2^) and LED@405 nm (60 mW/cm^2^) irradiation for 600s in the presence of **HNQA** (or **HNQE**) (0.5 wt%) or **HNQA** (or **HNQE**)/Iod (0.5%/2%, *w*/*w*).

	Acrylate Conversions (%)		100xRp/[M0] (s^−1^)	
Photoinitiating Systems	LED@385 nm	LED@405 nm	385 nm	405 nm
HNQE/SOA	51	63	0.5	1.3
HNQE/Iod/SOA	81	83	5	5.7
HNQ/SOA	-	-	-	-
HNQ/Iod/SOA	-	-	-	-
HNQA/SOA	30	34	0.5	0.9
HNQA/Iod/SOA	62	76	3.8	3.3

**Table 6:** Vinyl, thiol, and epoxy final conversions (determined by IR) after 600s of irradiation of limonene1,2 epoxide/trithiol (50 wt %/50 wt%) blend mixture under LED@385nm (44 mW/cm^2^) and LED@405nm (60 mW/cm^2^) irradiation under air and in laminate. Photoinitiating systems = **HNQA**/Iod (0.5/2%, *w*/*w*) and **HNQE**/Iod (0.5/2%, *w*/*w*).

	Vinyl Conversions (%)		Thiol Conversions (%)		Epoxy Conversions (%)	
Photosensitive Formulations	LED@ 385 nm	LED@ 405 nm	LED@ 385 nm	LED@ 405 nm	LED@ 385 nm	LED@405 nm
HNQA/Iod/(limonene 1,2 epoxide/trithiol)	96 ^a^ 98 ^b^	98 ^a^ 98 ^b^	43 ^a^ 64 ^b^	47 ^a^ 53 ^b^	30 ^a^	16 ^a^
HNQE/Iod/(limonene1,2 epoxide/trithiol)	98 ^a^ 99 ^b^	100 ^a^ 100 ^b^	53 ^a^ 62 ^b^	59 ^a^ 58 ^b^	-	-

^a^ under air, ^b^ in laminate.

## Data Availability

Data sharing not applicable.

## Supplementary Materials

The following are available online at https://www.mdpi.com/article/10.3390/polym13122015/s1, Figure S1: 1H NMR spectrum of HNQA, Figure S2: 13C NMR spectrum of HNQA, Figure S3, 1H NMR spectrum of HNQE, Figure S4: 13C NMR spectrum of HNQE, Figure S5: Molar extinction coefficient of 1) HNQ, 2) HNQE and 3) HNQA in ACN, Figure S6: Fluorescence quenching of HNQA upon gradual addition of Iod in ACN. Insert: Stern-Volmer plot I0/I as a function of the concentration of Iod for the calculation of the fluorescence quenching constants KSV, Figure S7: Fluorescence quenching of HNQE upon gradual addition of Iod in ACN. Insert: Stern-Volmer plot I0/I as a function of the concentration of Iod for the calculation of the fluorescence quenching constants KSV, Figure S8: Cyclic voltammogram of HNQA in ACN + 10^−3^ M tetraethylammonium tetrafluoroborate measured at a scan rate of 100 mV/s. [HNQA] = 10^−3^ M, Figure S9: Cyclic voltammogram of HNQE in ACN + 10-3 M tetraethylammonium tetrafluoroborate measured at a scan rate of 100 mV/s. [HNQE] = 10-3 M, Figure S10: Steady-state photolysis of HNQA/Iod/rhodamine B in ACN after irradiation by LED@405 nm (60 mW/cm^2^) under air conditions. [HNQA] = 5 × 10^−4^ M, [Iod] = 1.7 × 10^−3^ M and [RhB] = 3.10^−3^ M.
