# Supplementary material for: Lawsone Derivatives as Efficient Photopolymerizable Initiators for Free-Radical, Cationic Photopolymerizations, and Thiol—Ene Reactions
Source: Polymers (Basel). 2021 Jun 20;13(12):2015. doi: 10.3390/polym13122015 (PMC8234034; doi:10.3390/polym13122015)
Supplement: Supplementary file 1 [file polymers-13-02015-s001.zip › polymers-1260417-supplementary.pdf]

Supporting Information

Lawsone Derivatives as Efficient Photopolymerizable Initiators for Free-radical, Cationic Photopolymerizations and Thiol-ene Reactions

Christine Elian[1], Vlasta Brezova[2], Pauline Sautrot-Ba[1], Martin Breza[2], Davy-Louis Versace[1]

[1]Université Paris-Est Créteil (UPEC), ICMPE–UMR-CNRS 7182, 2-8 rue Henri Dunant, 94320 Thiais, France.

[2]Slovak University of Technology in Bratislava, Institute of Physical Chemistry and Chemical Physics, Department of Physical Chemistry, Radlinského 9, SK-812 37 Bratislava, Slovak Republic.

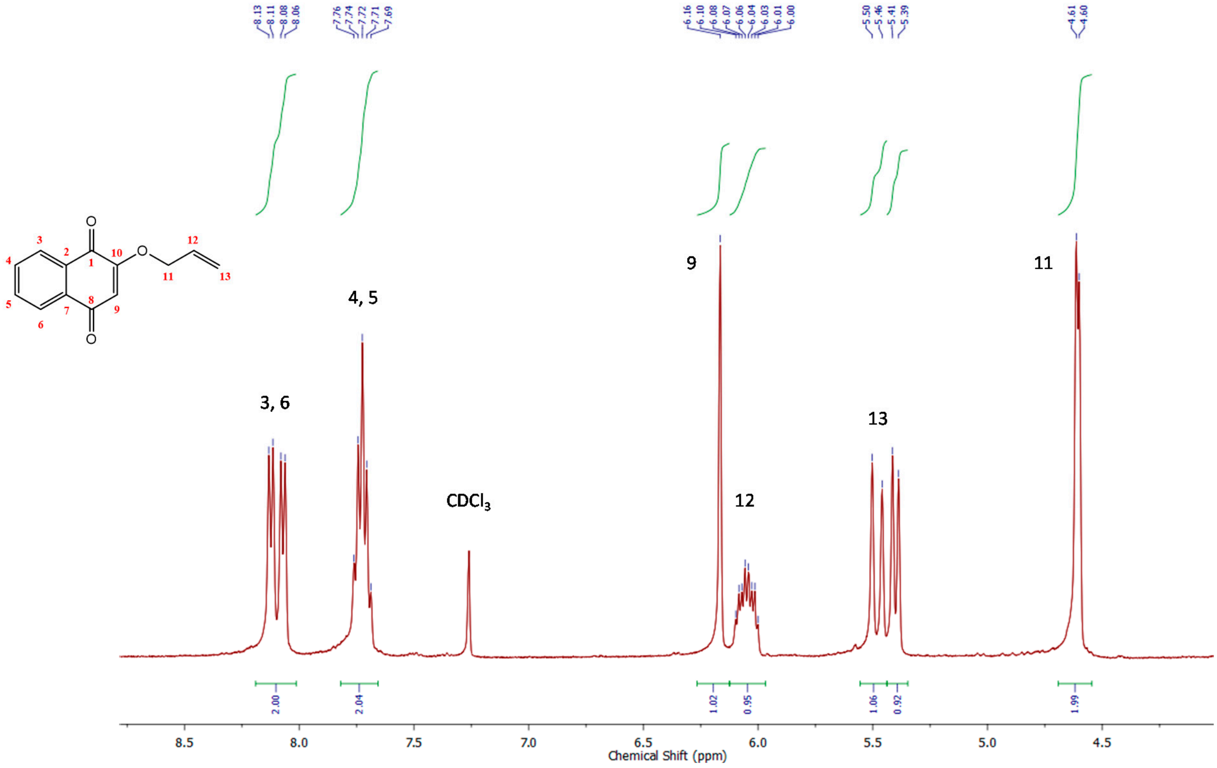

Figure S1. ^{1}H NMR spectrum of **HNQA**.

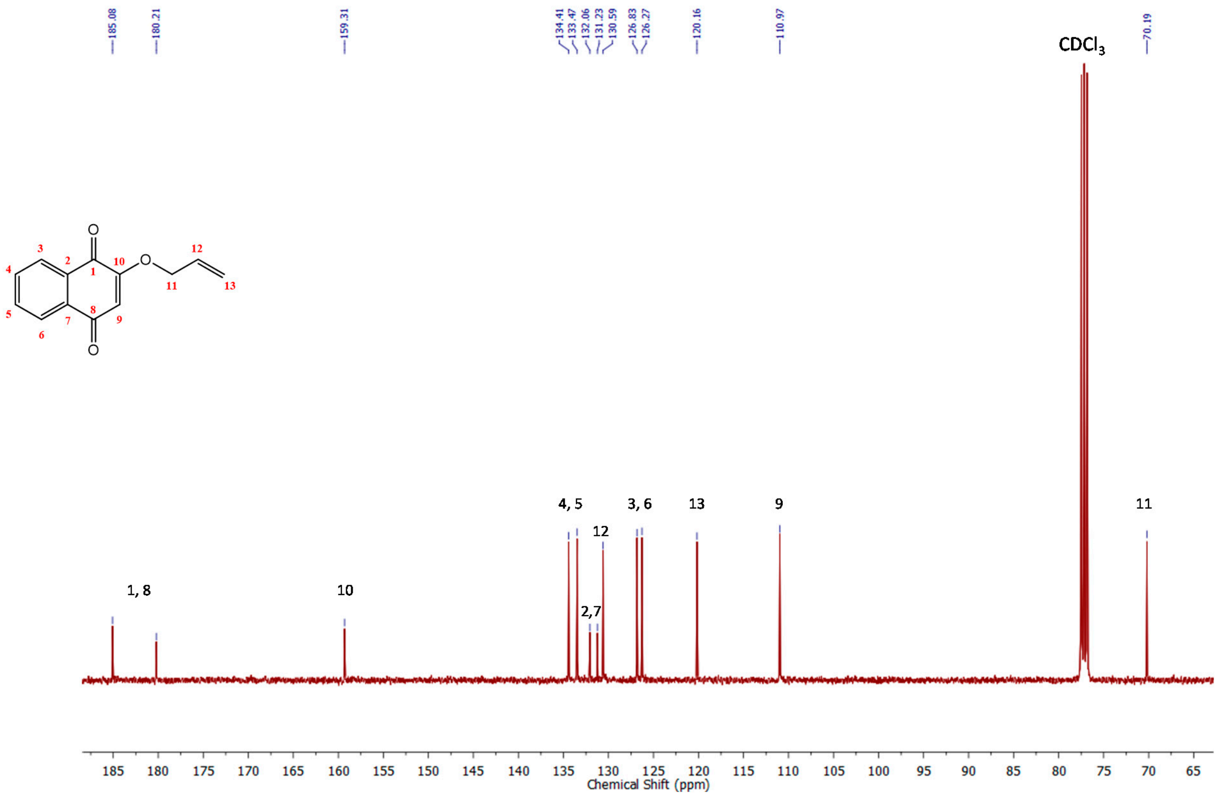

Figure S2. ^{13}C NMR spectrum of **HNQA**.

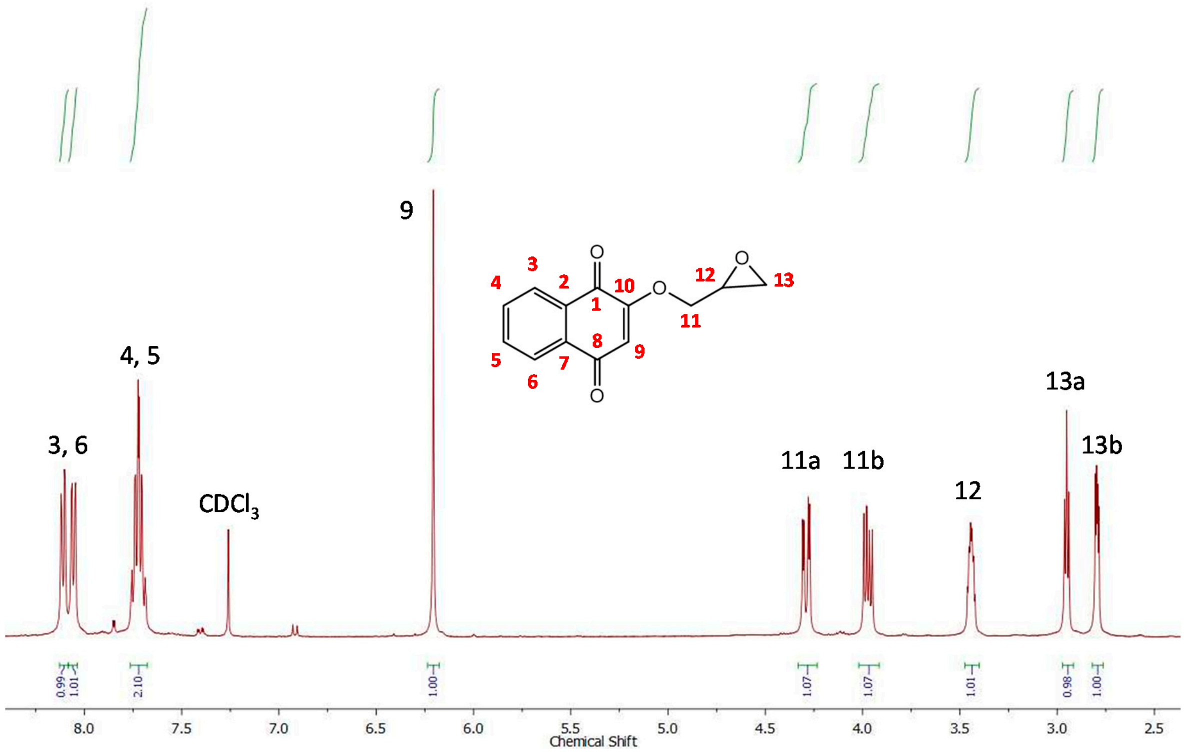

Figure S3. ^{1}H NMR spectrum of **HNQE**.

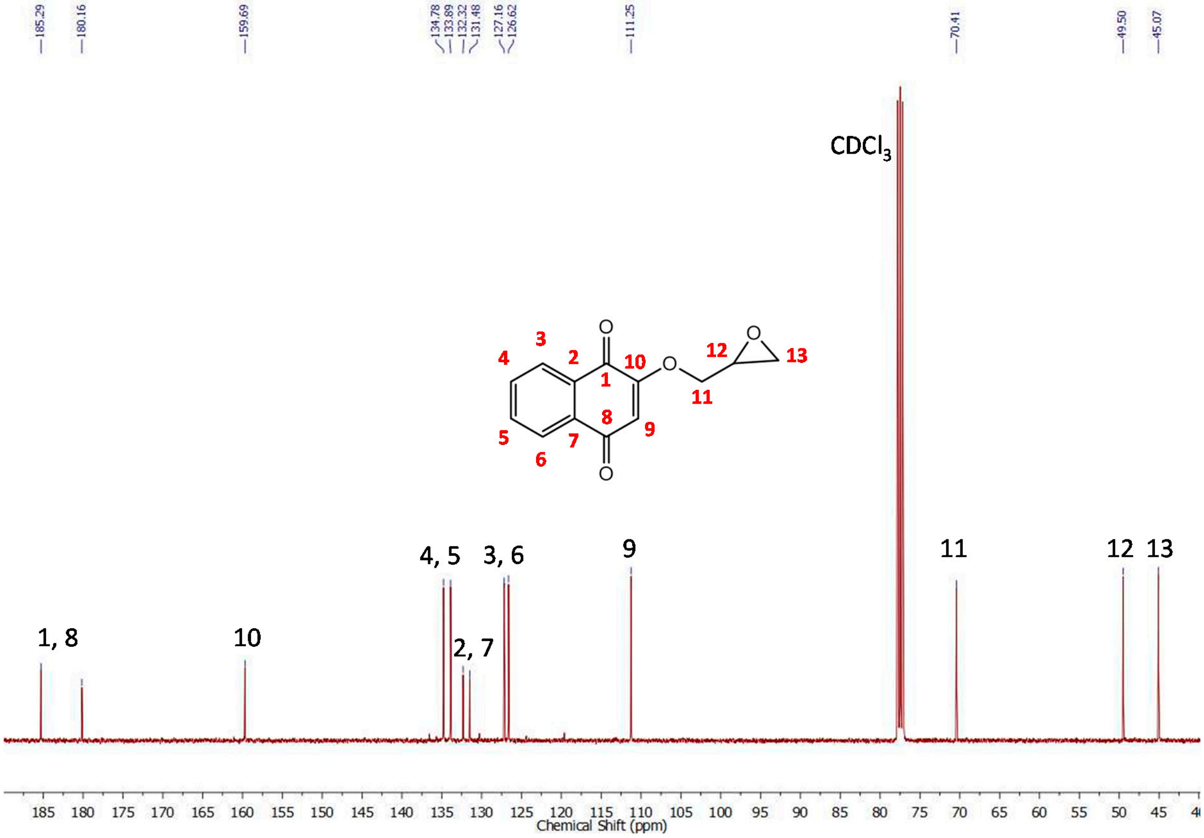

Figure S4. ^{13}C NMR spectrum of **HNQE**.

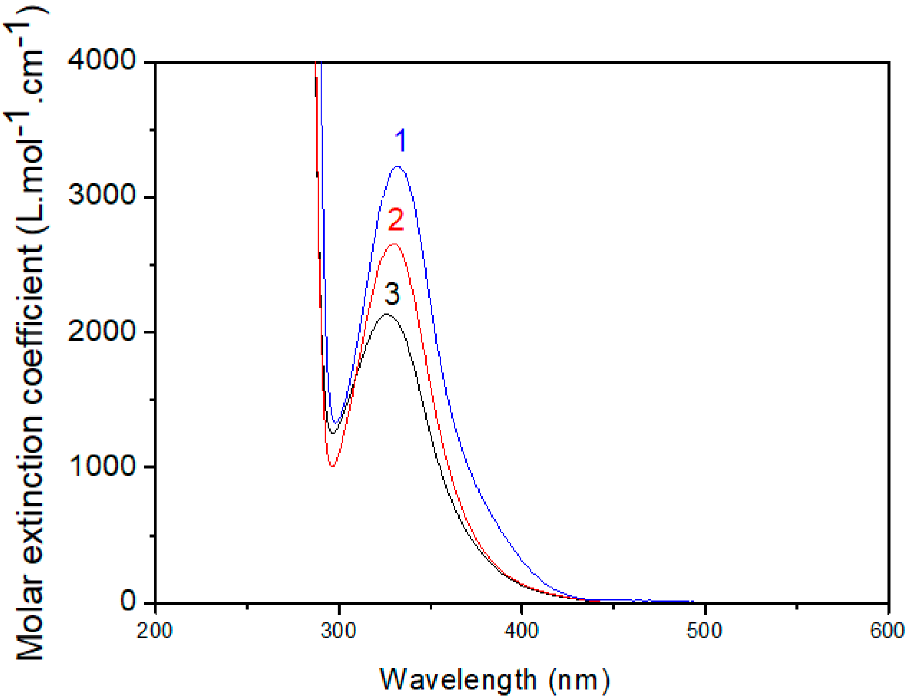

Figure S5. Molar extinction coefficient of 1) HNQ, 2) **HNQE** and 3) **HNQA** in ACN.

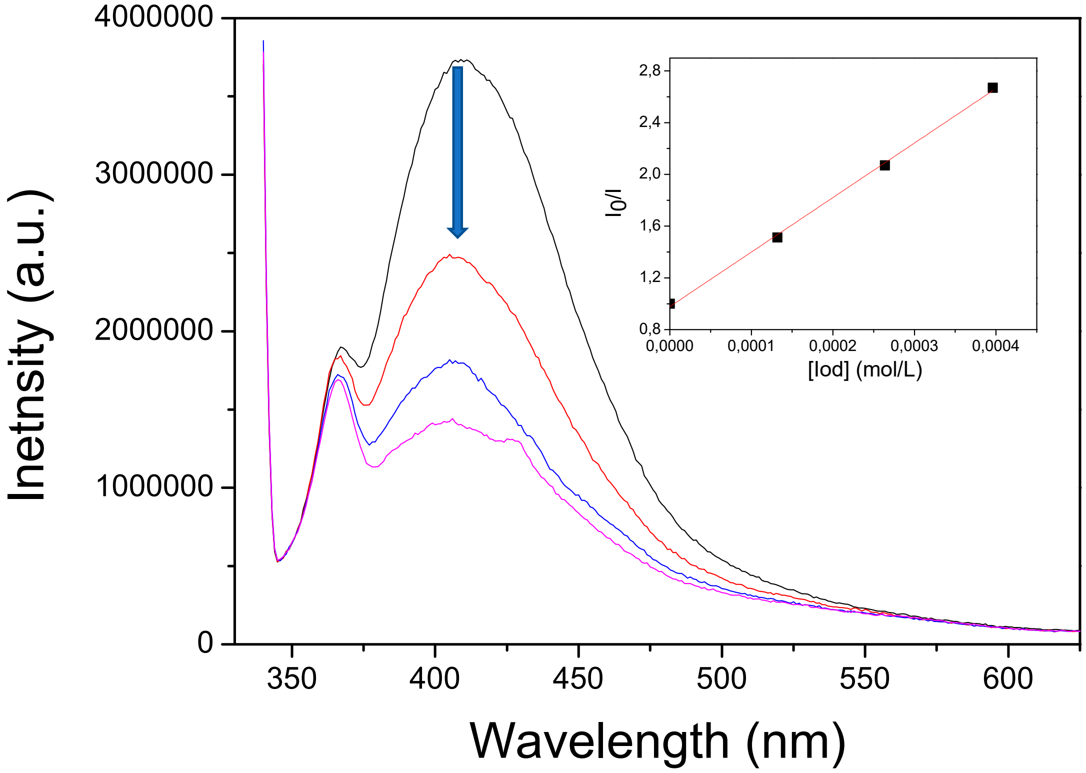

Figure S6. Fluorescence quenching of **HNQA** upon gradual addition of Iod in ACN. **Insert**: Stern-Volmer plot I_0/I as a function of the concentration of Iod for the calculation of the fluorescence quenching constants K_{SV}.

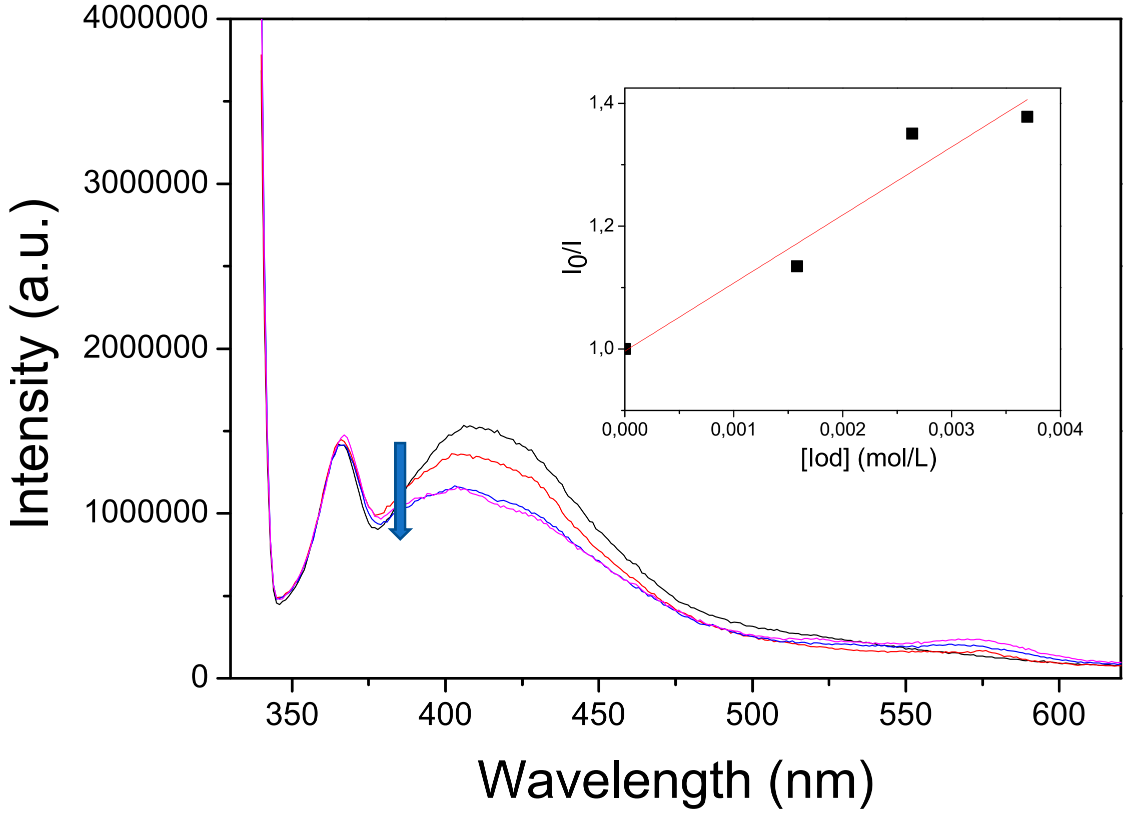

Figure S7. Fluorescence quenching of **HNQE** upon gradual addition of Iod in ACN. **Insert**: Stern-Volmer plot I_0/I as a function of the concentration of Iod for the calculation of the fluorescence quenching constants K_{SV}.

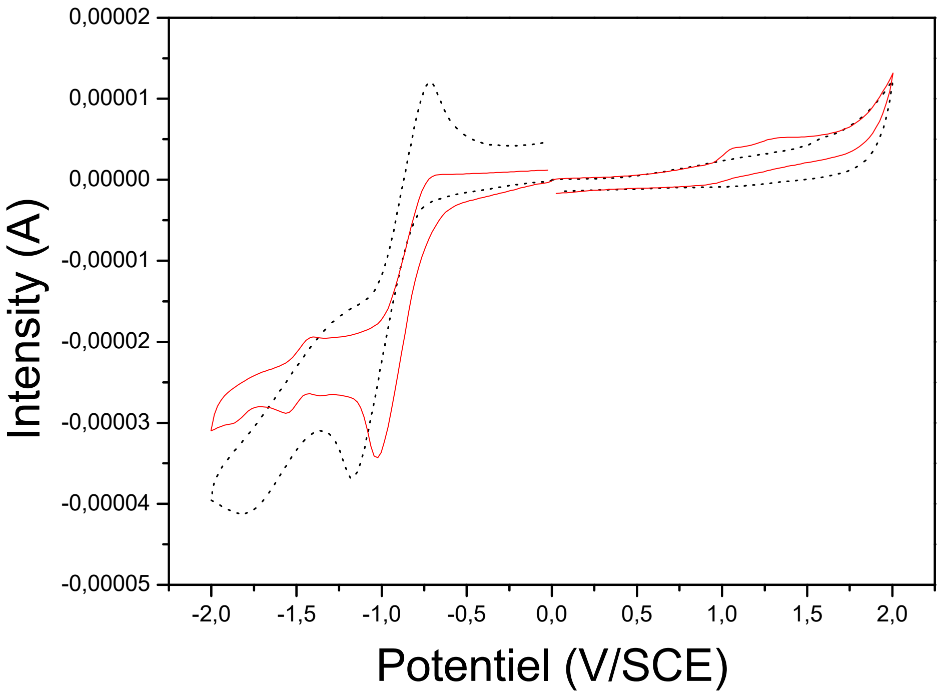

Figure S8. Cyclic voltammogram of **HNQA** in ACN + 10^{-3} M tetraethylammonium tetrafluoroborate measured at a scan rate of 100 mV/s. [**HNQA**] = 10^{-3} M.

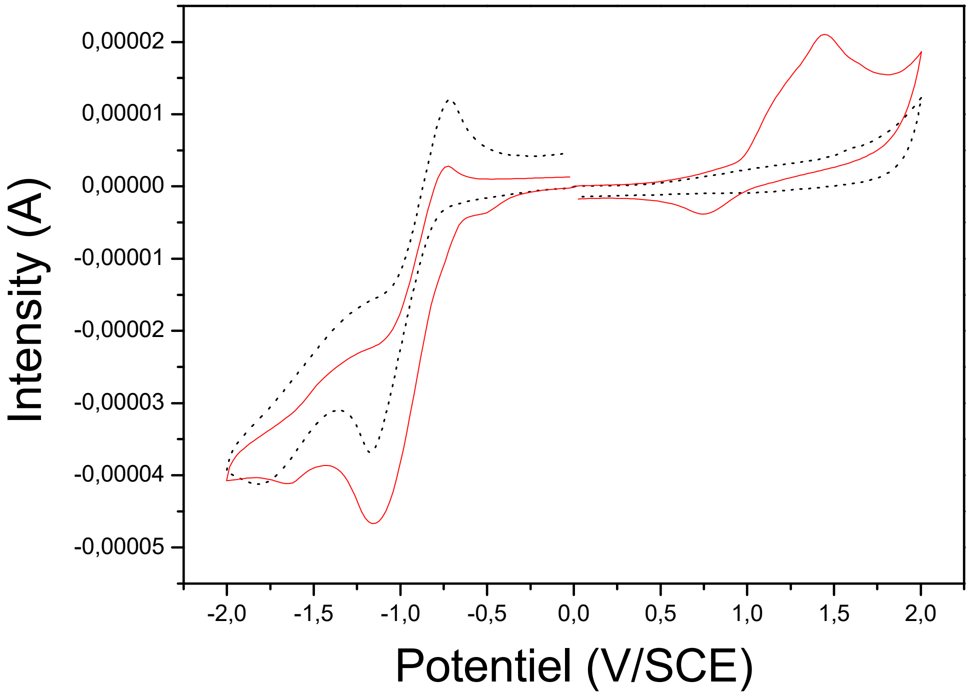

Figure S9. Cyclic voltammogram of **HNQE** in ACN + 10^{-3} M tetraethylammonium tetrafluoroborate measured at a scan rate of 100 mV/s. [**HNQE**] = 10^{-3} M.

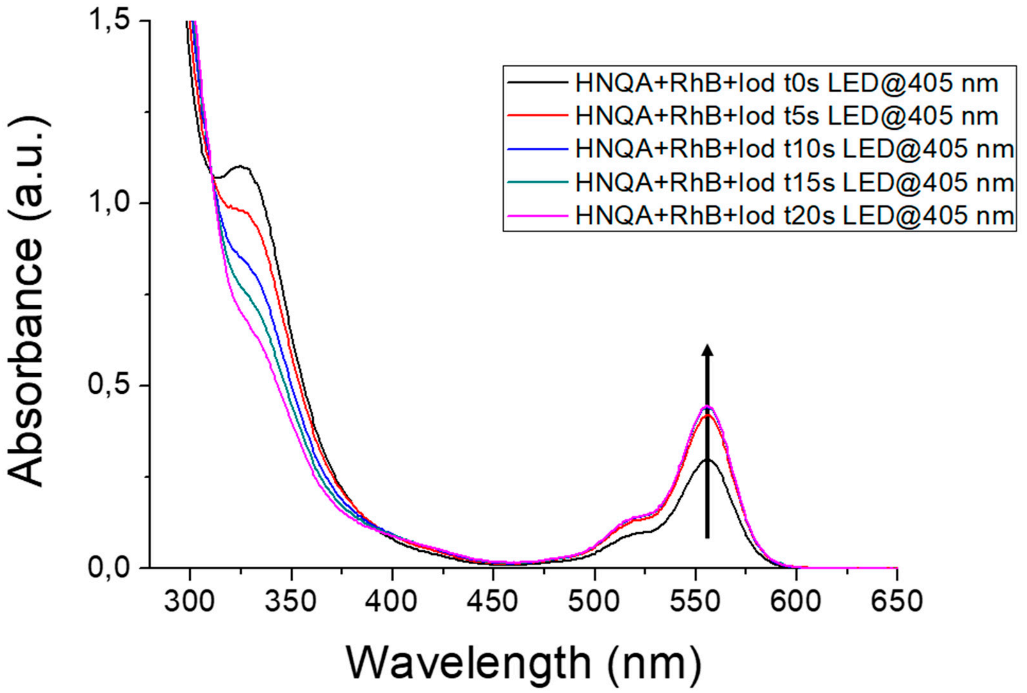

Figure S10. Steady-state photolysis of **HNQA**/Iod/rhodamine B in ACN after irradiation by LED@405 nm (60 mW/cm^2) under air conditions. [**HNQA**] = 5×10^{-4} M, [Iod] = 1.7×10^{-3} M and [RhB] = 3.10^{-6} M.
